# Cost-Effectiveness of Alirocumab for the Secondary Prevention of Cardiovascular Events after Myocardial Infarction in the Chinese Setting

**DOI:** 10.3389/fphar.2021.648244

**Published:** 2021-04-14

**Authors:** Zhe Liang, Qi Chen, Ruiqi Wei, Chenyao Ma, Xuehui Zhang, Xue Chen, Fang Fang, Quanming Zhao

**Affiliations:** ^1^ Department of Cardiology, Beijing Anzhen Hospital, Capital Medical University, Beijing, China; ^2^ Department of Sleep Medical Center, Beijing Anzhen Hospital, Capital Medical University, Beijing, China; ^3^ Department of Pulmonary and Critical Care Medicine, Beijing Anzhen Hospital, Capital Medical University, Beijing, China; ^4^ Department of Ultrasound, Beijing Ditan Hospital, Capital Medical University, Beijing, China

**Keywords:** alirocumab, cost-effectiveness, myocardial infarction, PCSK9 inhibitors, secondary prevention

## Abstract

**Background:** Proprotein convertase subtilisin/kexin type 9 inhibitor alirocumab reduce ischemic events; however, the cost-effectiveness remains uncertain. This study sought to evaluate its economic value in patients with myocardial infarction (MI) from the Chinese healthcare perspective.

**Methods:** A state-transition Markov model was developed to determine the cost-effectiveness of alirocumab for preventing recurrent MI, ischemic stroke and death. Preventative effect of the therapy was gathered from ODYSSEY OUTCOMES trial and absolute reduction of low-density lipoprotein cholesterol (LDL-C) in ODYSSEY EAST trial, respectively. The primary outcome was the incremental cost-effectiveness ratio (ICER), defined as incremental cost per quality-adjusted life-year (QALY) gained.

**Results:** Compared with statin monotherapy, the ICER of alirocumab therapy at its present discounted price [34,355 Chinese yuan (CNY) annually, 33% rebate] based on clinical follow-up efficacy was 1,613,997 CNY per QALY gained. A willingness-to-pay threshold of 212,676 CNY per QALY would be achieved when the annual cost of alirocumab was reduced by 88% from the full official price to 6071 CNY. The therapeutic effect evaluation estimated by the magnitude of LDL-C reduction was superior to the results of clinical follow-up, but this medication was still far from cost-effective. Multiple vulnerable subgroup analyses demonstrated that the ICER for patients with polyvascular disease in 3 vascular beds was 111,750 CNY per QALY gained.

**Conclusion:** Alirocumab is not cost-effective in general MI population based on current discounted price. High long-term costs of alirocumab may be offset by health benefit in patients with polyvascular disease (3 beds).

## Introduction

Cardiovascular disease (CVD) plays an increasing role in years of life lost currently, accounting for 40% of deaths in the Chinese population (Zhao et al., 2019). Low-density lipoprotein cholesterol (LDL-C) is a modifiable risk factor for CVD and the effective reduction of LDL-C benefit cardiovascular events (Stevens et al., 2016). High-intensity or maximally tolerated lipid-lowering therapy, especially in high-risk myocardial infarction (MI) individuals, is recommended in recent guidelines for lipid management (Mach et al., 2020).

In daily clinical practice, approximately 82.9% of MI patients, from a nationwide Swedish survey, would be eligible for intensive lipid-lowering therapy as not attaining the updated target of LDL-C level (Allahyari et al., 2020) which partly attributes to inefficiency, intolerance and non-adherence of statins (Soran et al., 2020). Innovation in the field of hyperlipemia sheds light on unmet needs. Alirocumab is a fully human monoclonal antibody biological medication that inhibits proprotein convertase subtilisin/kexin type 9 (PCSK9). It has showed powerful effect and safety in lipid-lowering and cardiovascular outcomes improvement in patients with heterozygous familial hypercholesterolemia (HeFH) or high cardiovascular risk (Kastelein et al., 2015; Farnier et al., 2016). In 2020, alirocumab was approved in priority by Chinese National Medical Products Administration based on Alirocumab and Cardiovascular Outcomes after Acute Coronary Syndrome (ODYSSEY OUTCOMES) trial (Schwartz et al., 2018). This landmark trial demonstrated that the risk of composite primary endpoints reduced by 15% among patients with previous acute coronary syndrome in alirocumab therapy compared with placebo.

Accurate economic evaluation of new therapy is available and necessary to make certain the treatment effect and potential tradeoffs among therapies after the results of the related large randomized controlled trials are released together with the price determined in healthcare system. Although the economic value of alirocumab is inconsistent in the published papers from American groups, substantial price reduction would definitely improve cost-effectiveness regardless of simulated methods (Kazi et al., 2016; Kazi et al., 2019; Bhatt et al., 2020). In consideration of the gap in healthcare system and economic status between US and China, there may be divergence in the aspect of cost-effectiveness of alirocumab in Chinese patients. Therefore, this study aimed to assess the value of alirocumab in clinical MI cohort under a long-term cost-effectiveness analysis from the Chinese healthcare perspective, which may guide policymakers, payers, clinicians and patients to have more precise price expectations. At the same time, high-risk subgroups were further screened to maximize the application value of alirocumab. This study is the continued section of our previous work (Liang et al., 2020).

## Methods

### Model Overview

**FIGURE 1:**
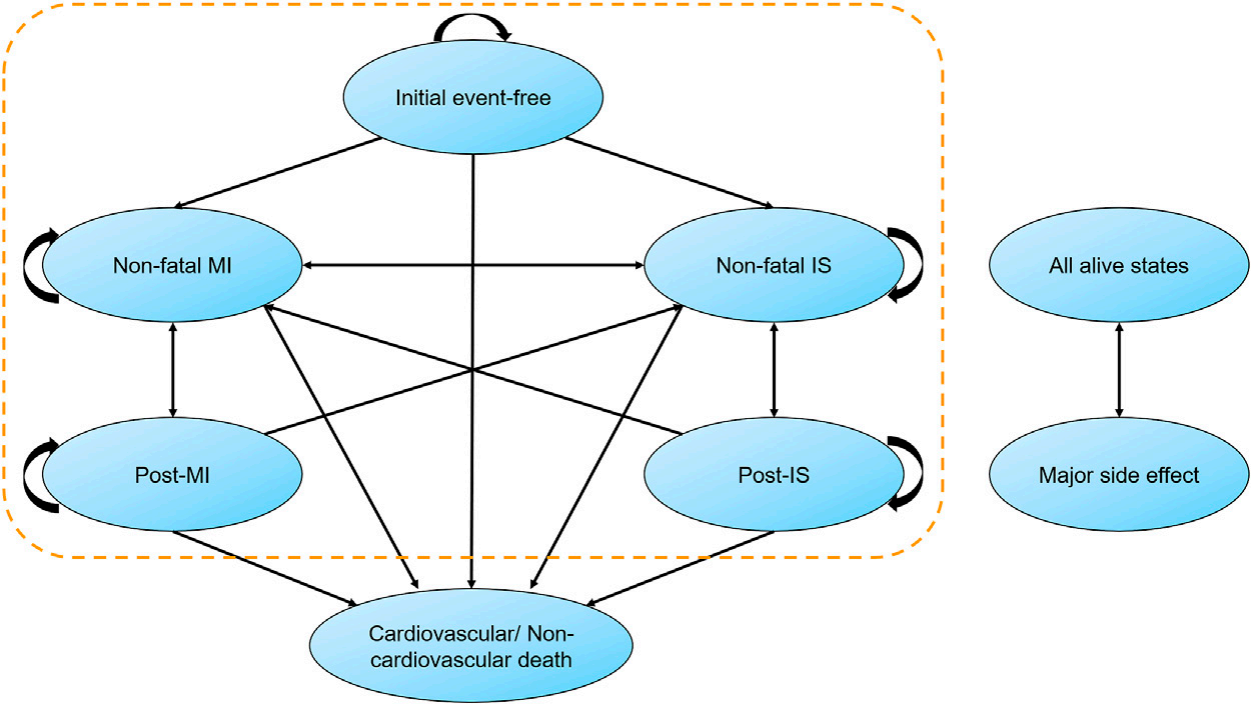
Markov state-transition model with health states and possible transitions. Patients could stay in their original state or convert to another state in each cycle. Major side effect was mainly defined as mild injection-site adverse reaction, which may occur at any time. IS, ischemic stroke; MI, myocardial infarction.

We established a state-transition Markov model to assess the economic value of alirocumab within established MI population (Figure 1). After a discharge of MI, patients entered the model as an initial event-free state, and the entire cohort was redistributed across states in every 1-year cycle. The model included mutually exclusive health states (non-fatal MI, non-fatal ischemic stroke [IS] and death). The major side effect (local injection-site reaction) was incorporated into the model. The time frame was 25 years in basic analysis to take the majority of Chinese old patients into consideration. Half-cycle correction was used for all events in every year.

Alirocumab plus statin therapy (high-intensity or maximally tolerated) vs. statin monotherapy were projected through accrued simulation. The incremental cost-effectiveness ratio (ICER), a primary outcome, was calculated by incremental cost per quality-adjusted life-years (QALYs) gained. We assumed the cost-effectiveness threshold as 212,676 Chinese Yuan (CNY, 1 CNY = 0.1450 USD in 2019 (National Bureau of Statistics of China, 2019a)), which was three times of per capita gross domestic product (GDP) in 2019 (National Bureau of Statistics of China, 2019a), based on the China Guidelines for Pharmacoeconomic Evaluations (Wu et al., 2015).

### Target Population

A representative real-world cohort with established MI was modeled to reflect the Chinese high-risk CVD population. Patients were selected from the China PEACE Prospective AMI study (China Patient-centered Evaluative Assessment of Cardiac Events Prospective Study of Acute Myocardial Infarction) (Dreyer et al., 2019), the first large clinical outcomes study in MI population in China. A total of 3,415 patients with AMI were recruited from 53 hospitals located in 21 of 31 provinces. During the one-year follow-up period, main clinical outcomes contained non-fatal MI (1.7%), non-fatal IS (0.9%), cardiovascular death (2.2%) and all-cause death (3.1%). More detailed characteristics of this target population are showed in Supplementary Table S1.

### Therapeutic Effect and Event Rates

The effect of alirocumab has been much-discussed in previous research literatures. We aimed to use two approaches to incorporate the efficacy of alirocumab in this health economic model (Table 1). One was applying the hazard ratios (HRs) observed in ODYSSEY OUTCOMES (Schwartz et al., 2018) to all population directly, taking no account of LDL-C levels, which was preferred assumption in evidence-based intervention and most economic evaluations. To model the effect of alirocumab on non-fatal IS, we used outcome data from ODYSSEY OUTCOMES for an end point defined as “fatal or non-fatal IS.” We assumed that the reduction of all-cause mortality was mediated through the reduction in the cardiovascular death risk. The other was integrating more specific lipid-lowering efficacy of the PCSK9 inhibitor in Chinese population observed in ODYSSEY EAST study (Han et al., 2020) with its estimated effect on major adverse cardiovascular events (MACEs) based on a Cholesterol Treatment Trialists’ Collaboration (CTTC) meta-analysis of statins trials (Baigent et al., 2010), which was also suitable for other medications such as ezetimibe and PCSK9 inhibitors.

**TABLE 1:** Key input parameters.

Input	Base-case value	Range	Distribution	References
Population event rates, %				
Non-fatal MI	1.7	Age dependent	NA	Dreyer et al. (2019), National Health Commission of the People’s Republic of China (2019)
Non-fatal IS	0.9	Age dependent	NA	Dreyer et al. (2019), National Health Commission of the People’s Republic of China (2019)
Cardiovascular death	2.2	Age dependent	NA	Dreyer et al. (2019), National Health Commission of the People’s Republic of China (2019)
Non-cardiovascular death	0.9	Age dependent	NA	Dreyer et al. (2019), National Health Commission of the People’s Republic of China (2019)
Therapeutic effect based on clinical endpoints, HR				
Non-fatal MI	0.86	0.77–0.96	Log normal	Schwartz et al. (2018)
Non-fatal IS	0.73	0.57–0.93	Log normal	Schwartz et al. (2018)
Cardiovascular death	0.88	0.74–1.05^a^	Log normal	Schwartz et al. (2018)
Therapeutic effect based on LDL-C reduction, RR				
Non-fatal MI	0.57	0.53–0.63	Log normal	Han et al. (2020), Baigent et al. (2010)
Non-fatal IS	0.66	0.59–0.75	Log normal	Han et al. (2020), Baigent et al. (2010)
Cardiovascular death	0.77	0.71–0.83	Log normal	Han et al. (2020), Baigent et al. (2010)
Annual cost of drugs, CNY				
Alirocumab				
Full list price	51,532	NA	NA	Calculated
Discounted net price	34,355	NA	NA	Calculated
Ezetimibe	2,827	NA	NA	Calculated
Cost of cardiovascular events, CNY^b^				
Initial event-free	8,344	6,258–10,430	Log normal	Fu et al. (2020)
Non-fatal MI	71,030	53,272–88,787	Log normal	Wang et al. (2015)
Post-MI	8,344	6,258–10,430	Log normal	Fu et al. (2020)
Non-fatal IS	22,342	16,756–27,927	Log normal	Yin et al. (2018)
Post-IS	8,463	6,347–10,578	Log normal	Fu et al. (2020)
Death due to MI	87,756	65,817–109,695	Log normal	Wang et al. (2015)
Death due to stroke	59,025	44,269–73,781	Log normal	Yin et al. (2018)
Cardiovascular death^c^	77,811	58,358–97,263	Log normal	Wang et al. (2015), Yin et al. (2018)
Non-cardiovascular death	0	NA	NA	NA
Quality of life^d^				
Initial event-free	0.824	0.800–0.848	β	Matza et al. (2015)
Non-fatal MI	0.672	0.625–0.719	β	Matza et al. (2015)
Post-MI	0.824	0.800–0.848	β	Matza et al. (2015)
Non-fatal IS	0.327	0.264–0.390	β	Matza et al. (2015)
Post-IS	0.524	0.472–0.576	β	Matza et al. (2015)
Injection site adverse reactions	−0.0003	−0.002-0	β	Khazeni et al. (2009)

CNY, Chinese Yuan; HR, hazard ratio; IS, ischemic stroke; LDL-C, low-density lipoprotein cholesterol; MI, myocardial infarction; NA, not applicable; RR, relative risk.

^a^ There was numerical but not significant reduction in cardiovascular mortality.

^b^ All costs varied by ± 25% in the sensitivity analysis because confidence intervals were not available from the primary data sources and are presented in 2019 CNY.

^c^ Cost of cardiovascular death was the weighted average cost of fatal MI and fatal stroke.

^d^ Utility values varied by 95% confidence intervals in the sensitivity analysis.

We assumed that the initial conversion probabilities above were applied to the first year and increased in every succedent 5 years in line with the growth rates of real-time natural mortalities in all age groups in China Health Statistics Yearbook (National Health Commission of the People’s Republic of China, 2019). All 25-years event rates in the basic analysis are listed in Supplementary Table S2.

### Costs and Utilities

The setting of this economic evaluation was based on Chinese healthcare sector. The direct costs associated with MACEs and subsequent chronic treatment, including initial event-free state, were obtained from previous literatures (Wang et al., 2015; Yin et al., 2018; Fu et al., 2020) and adjusted for inflation to 2019 (National Bureau of Statistics of China, 2019b) (Table 1).

Currently, public and private medical insurances do not cover PCSK9 inhibitors. Following the usual therapeutic dose and frequency (75 mg/2 weeks), the annual official price of alirocumab was estimated to be 51,532 CNY per person. As the PCSK9 inhibitor manufacturers had offered an average rebate of 33% since April 2020, main base case analysis was performed for discounted net price of alirocumab in 34,355 CNY annually. We also calculated the average bidding price of ezetimibe (10 mg), equally 2827 CNY per year, for scenario analyses.

Quality-of-life (QOL) estimates for health states were derived from a time tradeoff study (Matza et al., 2015). The discrepancy of mild injection-site adverse reaction (3.8% in alirocumab vs. 2.1% in placebo) was taken into account with a small penalty in QOL (Khazeni et al., 2009) and there was no increase in costs or therapy discontinuation. Future costs and QOL were discounted at 5.0% per year (Wu et al., 2015).

### Sensitivity Analyses

In one-way sensitivity analyses, we varied single input through plausible range (e.g., 95% confidence interval [CI]) (Table 1), while holding others at their base case values to search for key determinants to the ICERs. In probabilistic sensitivity analyses, multiple input parameters were randomly selected according to their pre-specified statistical distributions and generated 10,000 individual outputs surrounding the mean point estimate. The percentages of simulations falling below various willingness-to-pay (WTP) thresholds were drawn as cost-effectiveness acceptability curves.

### Scenario Analyses

Based on the ODYSSEY OUTCOMES trial, we made different hypothesis about effect of alirocumab. The efficacy of alirocumab on all-cause death (HR: 0.85; 95% CI: 0.73–0.98) was applied instead of the efficacy of alirocumab just on cardiovascular death in the base case. We modeled ezetimibe vs. alirocumab based on statin therapy. The efficacy of ezetimibe was modeled according to the IMPROVE-IT trial (Cannon et al., 2015) and the decrease of LDL-C (Baigent et al., 2010).

A range of scenario analyses were performed in high-risk subgroups to identify preferred candidates for the PCSK9 inhibitor. Women in China had higher all-cause/cardiovascular mortalities vs. men at 1-year post-MI (Dreyer et al., 2019), so we explored the value of alirocumab in female population. We involved poorly controlled FH patients after MI and assumed that the risk of MACEs was 2.3 times than those without FH (Wang et al., 2019). Meanwhile, a maximal reduction in risk of MACEs was modeled by the absolute change of LDL-C (Baigent et al., 2010). We also focused on patients with polyvascular disease in 3 vascular beds (MI with both peripheral artery disease and cerebrovascular disease) (Subherwal et al., 2012). In the ODYSSEY OUTCOMES research, a more pronounced absolute risk reduction was observed in patients with polyvascular disease than monovascular disease (Jukema et al., 2019). As the traditional risk factors for adverse events, cost-effectiveness of alirocumab were calculated in pre-existing diabetes mellitus (DM) (Li et al., 2016; Zhuo et al., 2019) and hypertension population (Chen et al., 2009; De Luca et al., 2014) with MI, respectively. Other scenario analyses were also performed, including initiating treatment among patients in different ages, varying the duration of alirocumab use between 5 and 30 years and applying various discount rates to the model.

We conducted all these analyses in disparate prices of alirocumab and simulation ways, if feasible, and key parameters are showed in Supplementary Table S3.

The model was performed by using TreeAge Pro 2020 R1.1 software program (TreeAge Software, Williamstown, MA). We followed the Consolidated Health Economic Evaluation Reporting Standards (CHEERS) guidelines (Husereau et al., 2013) and the reporting checklist for economic evaluation of health interventions is showed in Supplementary Table S4.

## Results

### Base Case Analysis

In the main result applying the HRs observed in ODYSSEY OUTCOMES to the model, mean costs would be 871,321 CNY with alirocumab added to statin therapy and 400,705 CNY with mere statin therapy. The clinical benefit of alirocumab were 0.29 gained QALYs. Compared with statin monotherapy, alirocumab at its present price (34,355 CNY, 33% rebate) was estimated to produce an ICER of 1,613,997 CNY per QALY gained (Table 2). If, instead, a full list price of alirocumab was used (51,532 CNY), the ICER would rise to 2,465,017 CNY per QALY. The conventional WTP threshold would be achieved when the annual cost of alirocumab was reduced by 88% from the full official price to 6071 CNY. Another indirect use of relative risks (RRs) hypothesis of LDL-C reduction with alirocumab yielded more optimistic ICER as 805,795 CNY per QALY based on discounted price of alirocumab accompanied with a net value-based price of 11,861 CNY per year.

**TABLE 2:** Base-case cost-effectiveness results.

Treatment strategy	Cost, CNY		QALY		ICER, CNY	VBP, CNY^a^
	Total	Incremental	Total	Incremental		
Discounted net price, therapeutic effect based on HRs of clinical endpoints						
Statins therapy alone	400,705	NA	7.22	NA	NA	NA
Alirocumab added to statins therapy	871,321	470,616	7.51	0.29	1,613,997	6,071
Full list price, therapeutic effect based on HRs of clinical endpoints						
Statins therapy alone	400,705	NA	7.22	NA	NA	NA
Alirocumab added to statins therapy	1,119,465	718,760	7.51	0.29	2,465,017	6,071
Discounted net price, therapeutic effect based on RRs of LDL-C reduction						
Statins therapy alone	400,705	NA	7.22	NA	NA	NA
Alirocumab added to statins therapy	842,176	441,471	7.77	0.55	805,795	11,861
Full list price, therapeutic effect based on RRs of LDL-C reduction						
Statins therapy alone	400,705	NA	7.22	NA	NA	NA
Alirocumab added to statins therapy	1,090,320	689,615	7.77	0.55	1,258,721	11,861

CNY, Chinese Yuan; HR, hazard ratio; ICER, incremental cost-effectiveness ratio; LDL-C, low-density lipoprotein cholesterol; NA, not applicable; QALY, quality-adjusted life-year; RR, relative risk; VBP, value-based price

^a^ VBP was defined as an estimated expected price to meet the ICER of 212,676 CNY per QALY gained

### Sensitivity Analyses

**FIGURE 2:**
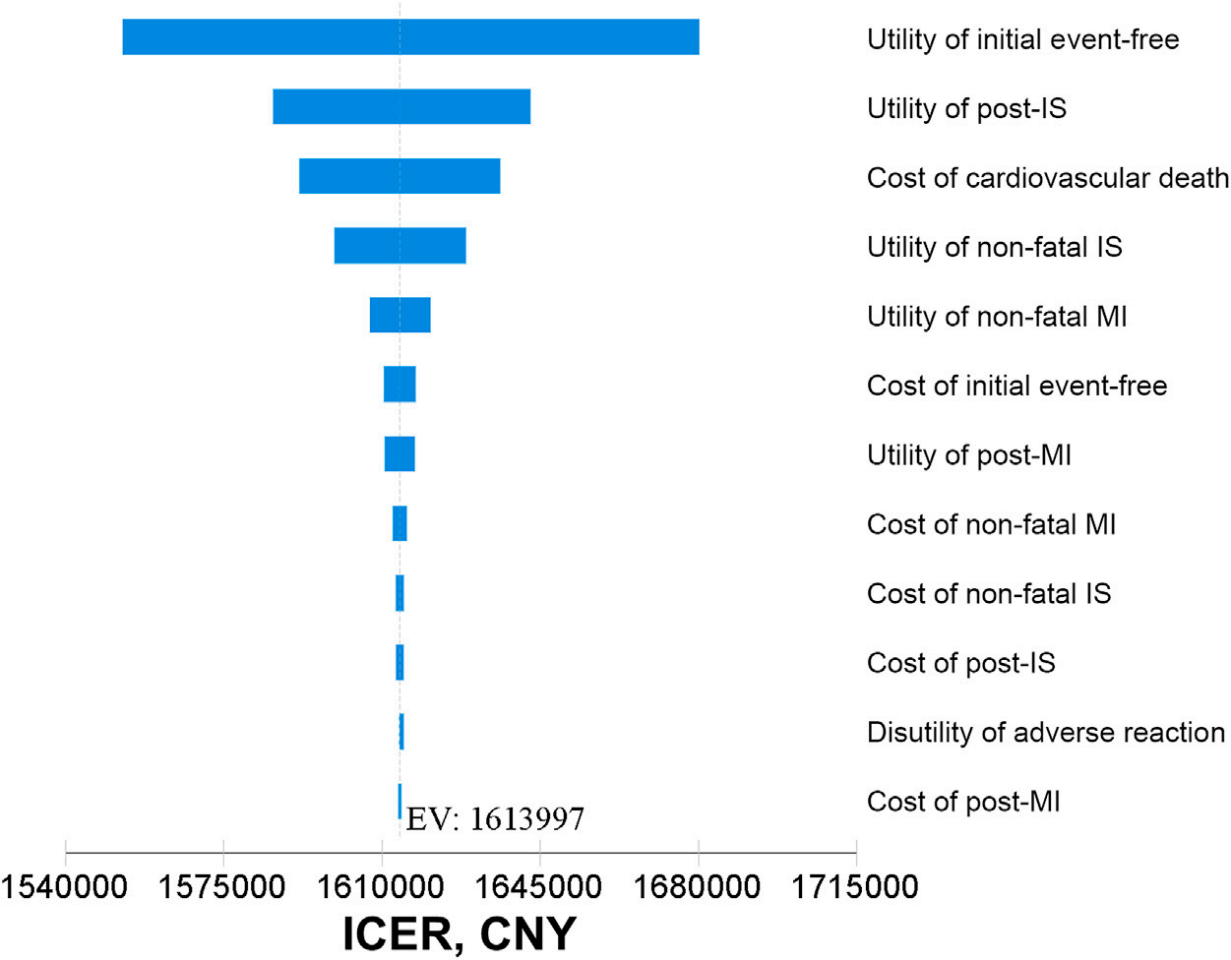
Tornado diagrams based on clinical follow-up efficacy at discounted price of alirocumab. CNY, Chinese Yuan; EV, expected value; ICER, incremental cost-effectiveness ratio; IS, ischemic stroke; MI, myocardial infarction.

In one-way sensitivity analyses, the main drivers on the ICER of alirocumab therapy at its present price based on clinical follow-up efficacy were the utility of initial event-free and post-IS (Figure 2). Additional tornado diagrams based on different evaluation ways and price points of alirocumab are presented in Supplementary Figure S1. The uncertain results for a wide range of assumptive inputs were still far beyond the WTP threshold, demonstrating the robustness and consistency of model outcomes.

**FIGURE 3:**
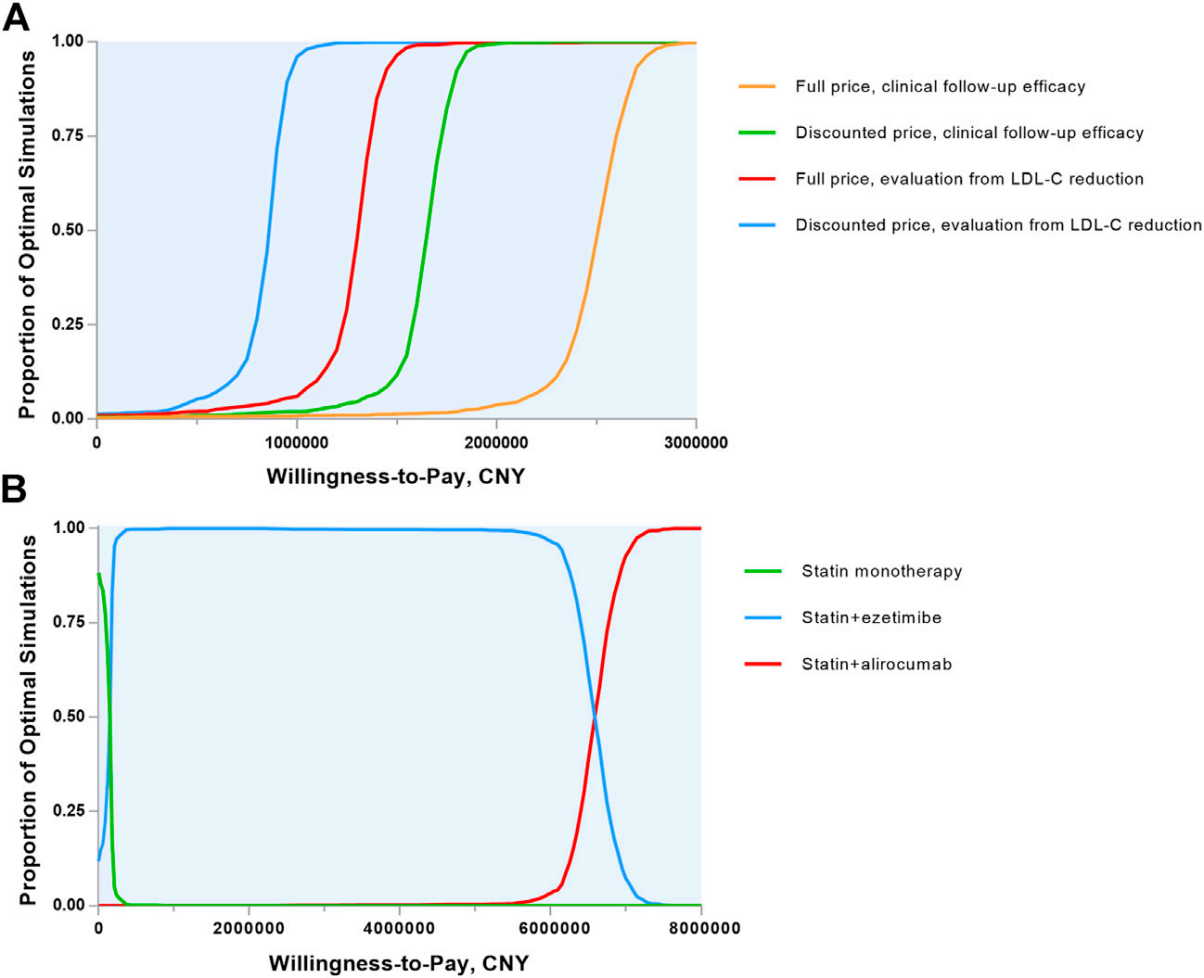
Cost-effectiveness acceptability curves. **(A)** Alirocumab plus statin therapy vs. statin monotherapy were compared and the 4 lines show the probability of alirocumab plus statin therapy to be cost-effective in a range of willingness-to-pay thresholds in various evaluation ways. **(B)** The 3 lipid-lowering strategies were included and the 3 lines shows the probability of each strategy to be cost-effective in a wide range of willingness-to-pay thresholds. CNY, Chinese Yuan; LDL-C, low-density lipoprotein cholesterol.

Probabilistic sensitivity analyses were demonstrated in Monte Carlo simulation scatters plots (Supplementary Figure S2) and cost-effectiveness acceptability curves (Figure 3A). Assuming the presupposed WTP threshold, the probability of alirocumab being cost-effective in MI population was 0.7% (0.3% for full list price) with clinical follow-up efficacy and 1.7% (1.1% for full list price) with LDL-C reduction hypothesis.

### Scenario Analyses

Separate scenarios were undertaken to evaluate the treatment benefit (Table 3). An all-cause death benefit yielded an ICER of 1,032,482 CNY and a value-based price of 8513 CNY annually. With the combination of ezetimibe and statin therapy, the ICER increased to 6,564,056 CNY and the cost of alirocumab must be reduced to 4232 CNY per year, which was unprecedented among biological medications in China. We also performed cost-effectiveness acceptability curves in three treatment ways (Figure 3B) and the main result showed that at the basic WTP threshold, the optimal treatment strategy was statin alone in 4.6%, statin plus ezetimibe in 95.2% and statin plus alirocumab in 0.2% of the simulation.

**TABLE 3:** Scenario analyses.

	Effect based on HRs of clinical endpoints			Effect based on RRs of LDL-C reduction		
Treatment alternative	ICER, CNY		VBP, CNY^a^	ICER, CNY		VBP, CNY^a^
	Discounted net price	Full list price		Discounted net price	Full list price	
Hypothesis of efficacy						
Reduction in non-cardiovascular death	1,032,482	1,577,392	8,513	860,508	1,323,098	10,300
Intervention therapy strategy						
Ezetimibe added to statins therapy group	6,564,056^b^	10,185,808^b^	4232^b^	1,229,700	1,953,885	10,232
Different subgroups						
Female population	1,777,745	2,722,174	5,890	858,907	1,348,791	11,696
FH With MI	NA	NA	NA	254,945	433,970	30,299
Polyvascular disease (3 beds)	111,750	217,596	50,734^c^	NA	NA	NA
DM with MI	1,364,704	2,092,499	7,165	673,573	1,061,929	13,970
Hypertension with MI	1,498,227	2,292,296	6,546	746,873	1,171,640	12,753
Starting age						
65 years	1,412,372	2,162,769	6,893	697,772	1,097,287	13,498
70 years	1,263,829	1,939,951	7,650	621,794	983,866	14,946
75 years	1,107,200	1,705,199	8,661	535,187	854,940	17,030
80 years	842,649	1,308,215	11,112	394,518	644,888	21,879
Time horizon						
5 years	4,849,493	7,314,287	2041	2,601,645	3,950,287	3,928
10 years	2,606,179	3,947,808	3,711	1,375,241	2,107,685	7,091
20 years	1,634,778	2,493,198	5,899	830,503	1,292,466	11,383
30 years	1,721,959	2,630,181	5,810	854,582	1,335,577	11,432
Discount rate						
3.5%	1,551,023	2,371,171	6,325	769,471	1,204,852	12,388
6%	1,659,693	2,533,180	5,900	831,970	1,297,604	11,510

CNY, Chinese Yuan; DM, diabetes mellitus; FH, familial hypercholesterolemia; HR, hazard ratio; ICER, incremental cost-effectiveness ratio; LDL-C, low-density lipoprotein cholesterol; MI, myocardial infarction; NA, not applicable; RR, relative risk; VBP, value-based price.

^a^ VBP was defined as an estimated expected price to meet the ICER of 212,676 CNY per QALY gained.

^b^ The effect of ezetimibe was modeled by integrating the clinical follow-up efficacy on non-fatal events and the assumed LDL-C reduction efficacy on cardiovascular death.

^c^ The discounted net price of alirocumab has achieved the willingness-to-pay threshold and a small reduction from the full list price was recommended.

Similar or slightly favorable economic value of the PCSK9 inhibitor was observed in several subgroups, including female, DM or hypertension with MI. For poorly controlled FH patients after MI, the ICER was 254,945 CNY per QALY gained and the price was expected to be 30,299 CNY, which was extremely close to the present acquisition price. In patients with polyvascular disease in 3 vascular beds, the ICER for alirocumab plus statin vs. statin alone was 111,750 CNY, which achieved the accepted WTP threshold. Meanwhile, the valued-based price was 50,734 CNY per year, just a small reduction from the full list price. Regarding to different initial treatment ages, the ICERs based on clinical follow-up efficacy declined from 1,412,372 CNY in 65 years old to 842,649 CNY in 80 years old. Long-term use of alirocumab (over 20 years) brought about relatively better economic benefit in general MI population. The diverse discount rates of 3.5% and 6% produced an annual expected price of 6325 CNY and 5900 CNY, respectively.

## Discussion

This is the first decision analysis to comprehensively assess the cost-effectiveness of alirocumab in China for our best knowledge. Despite the prominent cardiovascular benefit of alirocumab observed in the ODYSSEY OUTCOMES trial, its use was inefficient in Chinese healthcare system at present. The therapeutic effect evaluation estimated by the magnitude of LDL-C reduction was superior to the results of clinical follow-up, but this medication was still not cost-effective.

Two long-term economic assessments of alirocumab in US have been specially conducted. Kazi et al. (Kazi et al., 2019) simulated a cohort of US adults from a national survey and the original wholesale price of alirocumab would be reduced by 84% to meet the common WTP threshold. Conversely, Bhatt et al. (Bhatt et al., 2020) appeared to provide a more favorable viewpoint under ODYSSEY OUTCOMES patient-level cost-effectiveness analysis, especially yielding a high value among patients with baseline LDL-C over 100 mg/dl. The differences in baseline comorbid conditions, costs of cardiovascular care and prices of alirocumab in different periods accounted for the entirely disparate ICERs and more accurate and insightful analyses are expected to inform its potential cost consequences.

A noteworthy observation in the ODYSSEY OUTCOMES trial is the nominal reduction in all-cause mortality stratified from numerical reduction in cardiovascular and non-cardiovascular death. We assumed that the reduction of all-cause death was mediated through the reduction in the risk of cardiovascular death in main outcomes, which has an explicit mechanism induced by lip-lowering consisted with the CTTC meta-analysis (Baigent et al., 2010). However, there is also pathophysiological speculation that the benefit on non-fatal cardiovascular events may prevent disability, asthenia and susceptibility to non-cardiovascular illness and death (Steg et al., 2019). Therefore, we applied the overall HR for all-cause mortality to the model in scenario analyses and the ICER was improved but the conclusion was similar fundamentally. Patients with polyvascular disease in 3 vascular beds were only found likely to be cost-effective at present cost of alirocumab in multiple vulnerable subgroup analyses. Except the absolute risk increased in this group, a determining factor was the surprisingly low value of 0.23 observed for the HR for all cause death (Jukema et al., 2019). To our knowledge, this is also the first detailed cost-effectiveness simulation of PCSK9 inhibitors in polyvascular disease. There is no doubt that cardiac prevention in young group has more practical value than older counterpart from a clinical perspective. In the economic model, however, younger patients are low risk in MACEs and the widespread use of medication would not be offset by the benefit of reduced cardiovascular events, which accounts for why we recommend the long-term use of the PCSK9 inhibitor in older patients.

The economic value of evolocumab, another previously approved PCSK9 inhibitor in China, has been extensively studied and discussed in fully comparable methods (Liang et al., 2020). Despite evolocumab did not come out to be cost-effective in the general MI population as well, our analyses indicated that alirocumab was less cost-effective than evolocumab across main results and all subgroups. In addition to differences in actual prices of these agents, the somewhat different HRs concluded from ODYSSEY OUTCOMES (Schwartz et al., 2018) and FOURIER (Sabatine et al., 2017) trials play an indispensable role in evaluation process. In particular, evolocumab showed good value in MI patients with FH, whereas alirocumab did not. This is most likely due to our simulation of efficacy through LDL-C reduction, and the standard dose of evolocumab is slightly more effective than that of alirocumab. Korman et al. (Korman and Wisløff, 2018) drew the similar conclusion in the Norwegian setting. Due to lacking head-to-head comparison on clinical outcomes, there is no data to prove that one medication is superior to another yet. We evaluated their economic value based on the available data and do not recommend one over the other.

New expensive treatments which cure diseases or improve disability symptoms may be more compatible with their high prices. Delaying the process of atherosclerosis neither cure CVD nor reduce disability, so the potential benefits of PCSK9 inhibitors are limited. Less than one third of the patients in US complied with their prescriptions because of low approval rates or excessive out-of-pocket costs (Navar et al., 2017). There is lacking clinical data of the alirocumab application in Chinese patients; however, compared to other drugs supported by healthcare system, the high expense with self-paying of this medication apparently reduce the application adherence in real-world. With the progress of the development of several biotechnological agents except monoclonal antibodies targeted to PCSK9 protein (Jia et al., 2020) and the due expiration of PCSK9 inhibitors patent, more options will be available for the patients with lower price, which will bring expected improvement in the field of lipid-lowering therapy.

### Limitations

This study had several limitations. Our cohort selected from PEACE study had only one-year follow-up duration, which may overestimate the incidence of adverse events after MI and the ICERs would be even higher. In addition, the PEACE researchers did not describe the initial average LDL-C level of their cohort. To overcome the shortcomings, we used the absolute LDL-C reduction from the ODYSSEY EAST study involving Chinese population to simulate the effect of alirocumab. And we did not perform subgroup analyses in patients with baseline LDL-C level over or below 100 mg/dL for the above reason, just as a significant difference of therapeutic effect reported in ODYSSEY OUTCOMES trial. These limitations could not affect the conclusion that alirocumab was also not cost-effective in patients with initial LDL-C > 100 mg/dL accounted for the lack of economic value in MI patients with FH defined as higher LDL-C level over 200 mg/dL. In real-world clinical practice, the adherence of patients may decline based on over optimism of the condition or exorbitant costs. From an economic standpoint, there is no increase in cost or benefit when patients stop taking the medicine and therefore do not change the economic outcomes. If the legacy effect of PCSK9 inhibitors is proved, cost-effectiveness will likely rise. Lastly, our analysis is not generalizable to primary prevention or patients with stable CVD.

## Conclusion

At its current discounted price of 34,355 CNY annually, the addition of alirocumab to statin therapy in MI patients do not attain the generally accepted cost-effectiveness threshold. If pragmatic strategies can be carried out to control cost of alirocumab to 6071 CNY (assessed from clinical follow-up efficacy) or 11,861 CNY (assessed from LDL-C reduction), this agent would be a promising treatment option for high-risk patients. Expensive lifetime drug costs may be offset by clinical benefit in patients with polyvascular disease in 3 vascular beds.

## Data Availability

The original contributions presented in the study are included in the article/Supplementary Material, further inquiries can be directed to the corresponding authors.

## Abbreviations

**CVD**, cardiovascular disease; **LDL‐C**, low‐density lipoprotein cholesterol; **MI**, myocardial infarction; **PCSK9**, proprotein convertase subtilisin/kexin type 9; **HeFH**, heterozygous familial hypercholesterolemia; **IS**, ischemic stroke; **ICER**, incremental cost-effectiveness ratio; **QALY**, quality-adjusted life‐year; **CNY**, Chinese Yuan; **GDP**, gross domestic product; **HR**, hazard ratio; **MACE**, major adverse cardiovascular event; **CTTC**, cholesterol treatment trialists’ collaboration; **QOL**, quality‐of‐life; **CI**, confidence interval; **WTP**, willingness‐to‐pay; **DM**, diabetes mellitus; **CHEERS**, consolidated health economic evaluation reporting standards; **RR**, relative risk.
