# Supplementary material for: Cost-Effectiveness of Alirocumab for the Secondary Prevention of Cardiovascular Events after Myocardial Infarction in the Chinese Setting
Source: Front Pharmacol. 2021 Apr 14;12:648244. doi: 10.3389/fphar.2021.648244 (PMC8080443; doi:10.3389/fphar.2021.648244)
Supplement: Supplementary file 1 [file datasheet1.docx]

**SUPPLEMENTARY MATERIAL**

**Table S1 Baseline characteristics of the model population from clinical practice compared with patients enrolled in the ODYSSEY OUTCOMES trial**

| **Characteristics** | **Model population (n=3415)** | **ODYSSEY OUTCOMES trial population (n=18,924)** |
| --- | --- | --- |
| **Demographics** | | |
| Age, mean (SD), y | 60.7 (11.9) | 58.6 (9.4) |
| Female sex, No. (%) | 791 (23.2) | 4762 (25.2) |
| **Cardiovascular risk factors, No. (%)** | | |
| Diabetes mellitus | 794 (23.3) | 5444 (28.8) |
| Hypertension | 1899 (55.6) | 12,249 (64.7) |
| Current tobacco smoker | 1994 (58.4) | 4560 (24.1) |
| Body-mass index**^*^**, mean (SD), kg/m^2^ | 24.9 (4.0) | 28.5 (4.8) |
| **Medical history, No. (%)** | | |
| MI | 274 (8.0) | 3633 (19.2) |
| PCI or CABG | 241 (7.1) | 4288 (22.7) |
| Stroke | 564 (16.5) | 611 (3.2) |
| Peripheral artery disease | 11 (0.3) | 759 (4.0) |
| Congestive heart failure | 232 (6.8) | 2814 (14.9) |
| **Cardiovascular medications, No. (%)** | | |
| Statins | 2561 (75.0) | 18,464 (97.6) |
| Antiplatelet agent | | |
| Aspirin | 2616 (76.6) | 18,086 (95.6) |
| P2Y12 inhibitor | 2387 (69.9) | 16,541 (87.4) |
| Beta-blockers | 1827 (53.5) | 15,990 (84.5) |
| ACEIs or ARBs | 1448 (42.4) | 14,716 (77.8) |
| **MACEs per year, No. (%)** | | |
| Death from cardiovascular cause | 75 (2.2) | 183 (1.0) |
| Death from any cause | 105 (3.1) | 259 (1.4) |
| MI | 58 (1.7) | 481 (2.5) |
| IS | 30 (0.9) | 94 (0.5) |
| Coronary revascularization | 219 (6.4) | 557 (2.9) |

ACEI: angiotensin-converting enzyme inhibitor; ARB: angiotensin-receptor blocker; CABG: coronary artery bypass grafting; IS: ischemic stroke; MACE: major adverse cardiovascular event; MI: myocardial infarction; ODYSSEY OUTCOMES: Alirocumab and Cardiovascular Outcomes After Acute Coronary Syndrome; PCI: percutaneous coronary intervention; SD: standard deviation

**^*^** The body-mass index is the weight in kilograms divided by the square of the height in meters

**Table S2 Age-specific transition probabilities in the basic analysis**

| **Age in events, years** | **Alirocumab added to Statins therapy, %** | | **Statins therapy alone, %** |
| --- | --- | --- | --- |
|  | **Effect based on HRs of clinical endpoints** | **Effect based on RRs of LDL-C reduction** |  |
| **Non-fatal MI** | | | |
| 60-64 | 1.46 | 0.97 | 1.70 |
| 65-69 | 2.37 | 1.57 | 2.76 |
| 70-74 | 3.27 | 2.18 | 3.81 |
| 75-79 | 5.28 | 3.51 | 6.15 |
| 80-84 | 12.43 | 8.26 | 14.47 |
| Over 85 | 24.05 | 15.94 | 27.96 |
| **Non-fatal IS** | | | |
| 60-64 | 0.66 | 0.59 | 0.90 |
| 65-69 | 1.24 | 1.11 | 1.69 |
| 70-74 | 2.11 | 1.89 | 2.88 |
| 75-79 | 3.79 | 3.39 | 5.17 |
| 80-84 | 8.96 | 8.01 | 12.22 |
| Over 85 | 14.97 | 13.53 | 20.50 |
| **Cardiovascular death** | | | |
| 60-64 | 1.94 | 1.69 | 2.20 |
| 65-69 | 3.32 | 2.89 | 3.76 |
| 70-74 | 4.99 | 4.35 | 5.66 |
| 75-79 | 8.44 | 7.35 | 9.57 |
| 80-84 | 19.91 | 17.34 | 22.58 |
| Over 85 | 36.08 | 31.57 | 41.00 |
| **Non-cardiovascular death** | | | |
| 60-64 | 0.90 | 0.90 | 0.90 |
| 65-69 | 1.36 | 1.36 | 1.36 |
| 70-74 | 1.80 | 1.80 | 1.80 |
| 75-79 | 2.67 | 2.67 | 2.67 |
| 80-84 | 5.70 | 5.70 | 5.70 |
| Over 85 | 10.54 | 10.54 | 10.54 |

HR: hazard ratio; IS: ischemic stroke; LDL-C: low-density lipoprotein cholesterol; MI: myocardial infarction; RR: relative risk

**Table S3 Initial transition probabilities in scenario analyses**

| **Treatment alternative** | **Effect based on HRs of clinical endpoints** | | | **Effect based on RRs of LDL-C reduction** | | |
| --- | --- | --- | --- | --- | --- | --- |
|  | **Event rate, %** | | **HR** | **Event rate, %** | | **RR** |
|  | **Alirocumab added to Statins therapy** | **Statins therapy alone** |  | **Alirocumab added to Statins therapy** | **Statins therapy alone** |  |
| **Reduction in non-cardiovascular death** | | | | | | |
| Non-fatal MI | 1.46 | 1.70 | 0.86 | 0.97 | 1.70 | 0.57 |
| Non-fatal IS | 0.66 | 0.90 | 0.73 | 0.59 | 0.90 | 0.66 |
| All-cause death | 2.64 | 3.10 | 0.85 | 2.57 | 3.10 | 0.83 |
| **Ezetimibe added to Statins therapy group** | | | | | | |
| Non-fatal MI | 1.46 | 1.48 | 0.99 | 0.97 | 1.39 | 0.70 |
| Non-fatal IS | 0.66 | 0.71 | 0.93 | 0.59 | 0.77 | 0.76 |
| Cardiovascular death | 1.94 | 2.00 | 0.97 | 1.69 | 2.00 | 0.84 |
| **Female population** | | | | | | |
| Non-fatal MI | 1.72 | 2.00 | 0.86 | 1.14 | 2.00 | 0.57 |
| Non-fatal IS | 0.58 | 0.80 | 0.73 | 0.53 | 0.80 | 0.66 |
| Cardiovascular death | 3.61 | 4.10 | 0.88 | 3.16 | 4.10 | 0.77 |
| Non-cardiovascular death**^*^** | 1.80 | 1.80 | NA | 1.80 | 1.80 | NA |
| **FH with MI** | | | | | | |
| Non-fatal MI | NA | NA | NA | 1.21 | 3.91 | 0.31 |
| Non-fatal IS | NA | NA | NA | 0.85 | 2.07 | 0.41 |
| Cardiovascular death | NA | NA | NA | 2.88 | 5.06 | 0.57 |
| **Polyvascular disease (3 beds)** | | | | | | |
| Non-fatal MI | 1.59 | 2.45 | 0.65 | NA | NA | NA |
| Non-fatal IS | 0.71 | 1.30 | 0.55 | NA | NA | NA |
| Cardiovascular death | 0.78 | 3.28 | 0.24 | NA | NA | NA |
| **DM with MI** | | | | | | |
| Non-fatal MI | 2.44 | 2.84 | 0.86 | 1.62 | 2.84 | 0.57 |
| Non-fatal IS | 1.22 | 1.67 | 0.73 | 1.10 | 1.67 | 0.66 |
| Cardiovascular death | 3.68 | 4.18 | 0.88 | 3.22 | 4.18 | 0.77 |
| **Hypertension with MI** | | | | | | |
| Non-fatal MI | 1.94 | 2.26 | 0.86 | 1.29 | 2.26 | 0.57 |
| Non-fatal IS | 0.83 | 1.13 | 0.73 | 0.75 | 1.13 | 0.66 |
| Cardiovascular death | 2.46 | 2.79 | 0.88 | 2.15 | 2.79 | 0.77 |

DM: diabetes mellitus; FH: familial hypercholesterolemia; HR: hazard ratio; IS: ischemic stroke; LDL-C: low-density lipoprotein cholesterol; MI: myocardial infarction; NA: not applicable; RR: relative risk

**^*^** The non-cardiovascular mortality in female population was higher than male population, and the remaining unlisted non-cardiovascular mortalities in alternative scenarios were assumed to be consistent with the general population

**Table S4 Reporting checklist for economic evaluation of health interventions**

| **Element** | **Reporting Item** | **availability** |
| --- | --- | --- |
| **Title** | | |
|  | Identify the study as an economic evaluation or use more specific terms such as “cost-effectiveness analysis”, and describe the interventions compared. | Yes |
| **Abstract** | | |
|  | Provide a structured summary of objectives, perspective, setting, methods (including study design and inputs), results (including base case and uncertainty analyses), and conclusions. | Yes |
| **Introduction** | | |
| Background and objectives | Provide an explicit statement of the broader context for the study. Present the study question and its relevance for health policy or practice decisions. | Yes |
| **Methods** | | |
| Target population and subgroups | Describe characteristics of the base case population and subgroups analyzed, including why they were chosen. | Yes |
| Setting and location | State relevant aspects of the system(s) in which the decision(s) need(s) to be made. | Yes |
| Study perspective | Describe the perspective of the study and relate this to the costs being evaluated. | Yes |
| Comparators | Describe the interventions or strategies being compared and state why they were chosen. | Yes |
| Time horizon | State the time horizon(s) over which costs and consequences are being evaluated and say why appropriate. | Yes |
| Discount rate | Report the choice of discount rate(s) used for costs and outcomes and say why appropriate. | Yes |
| Choice of health outcomes | Describe what outcomes were used as the measure(s) of benefit in the evaluation and their relevance for the type of analysis performed. | Yes |
| Measurement of effectiveness | Single study-based estimates: Describe fully the design features of the single effectiveness study and why the single study was a sufficient source of clinical effectiveness data. | NA |
| Measurement of effectiveness | Synthesis-based estimates: Describe fully the methods used for identification of included studies and synthesis of clinical effectiveness data. | Yes |
| Measurement and valuation of preference-based outcomes | If applicable, describe the population and methods used to elicit preferences for outcomes. | Yes |
| Estimating resources and costs | Single study-based economic evaluation: Describe approaches used to estimate resource use associated with the alternative interventions. Describe primary or secondary research methods for valuing each resource item in terms of its unit cost. Describe any adjustments made to approximate to opportunity costs. | NA |
| Estimating resources and costs | Model-based economic evaluation: Describe approaches and data sources used to estimate resource use associated with model health states. Describe primary or secondary research methods for valuing each resource item in terms of its unit cost. Describe any adjustments made to approximate to opportunity costs. | Yes |
| Currency, price date, and conversion | Report the dates of the estimated resource quantities and unit costs. Describe methods for adjusting estimated unit costs to the year of reported costs if necessary. Describe methods for converting costs into a common currency base and the exchange rate. | Yes |
| Choice of model | Describe and give reasons for the specific type of decision analytical model used. Providing a figure to show model structure is strongly recommended. | Yes |
| Assumptions | Describe all structural or other assumptions underpinning the decision-analytical model. | Yes |
| Analytical methods | Describe all analytical methods supporting the evaluation. This could include methods for dealing with skewed, missing, or censored data; extrapolation methods; methods for pooling data; approaches to validate or make adjustments (such as half cycle corrections) to a model; and methods for handling population heterogeneity and uncertainty. | Yes |
| **Results** | | |
| Study parameters | Report the values, ranges, references, and, if used, probability distributions for all parameters. Report reasons or sources for distributions used to represent uncertainty where appropriate. Providing a table to show the input values is strongly recommended. | Yes |
| Incremental costs and outcomes | For each intervention, report mean values for the main categories of estimated costs and outcomes of interest, as well as mean differences between the comparator groups. If applicable, report incremental cost-effectiveness ratios. | Yes |
| Characterizing uncertainty | Single study-based economic evaluation: Describe the effects of sampling uncertainty for the estimated incremental cost and incremental effectiveness parameters, together with the impact of methodological assumptions (such as discount rate, study perspective). | NA |
| Characterizing uncertainty | Model-based economic evaluation: Describe the effects on the results of uncertainty for all input parameters, and uncertainty related to the structure of the model and assumptions. | Yes |
| Characterizing heterogeneity | If applicable, report differences in costs, outcomes, or cost effectiveness that can be explained by variations between subgroups of patients with different baseline characteristics or other observed variability in effects that are not reducible by more information. | Yes |
| **Discussion** | | |
| Study findings, limitations, generalizability, and current knowledge | Summarize key study findings and describe how they support the conclusions reached. Discuss limitations and the generalizability of the findings and how the findings fit with current knowledge. | Yes |
| **Other** | | |
| Source of funding | Describe how the study was funded and the role of the funder in the identification, design, conduct, and reporting of the analysis. Describe other non-monetary sources of support. | Yes |
| Conflict of interest | Describe any potential for conflict of interest of study contributors in accordance with journal policy. In the absence of a journal policy, we recommend authors comply with International Committee of Medical Journal Editors recommendations. | Yes |

NA: not applicable

**Figure S1 Tornado diagrams**

**
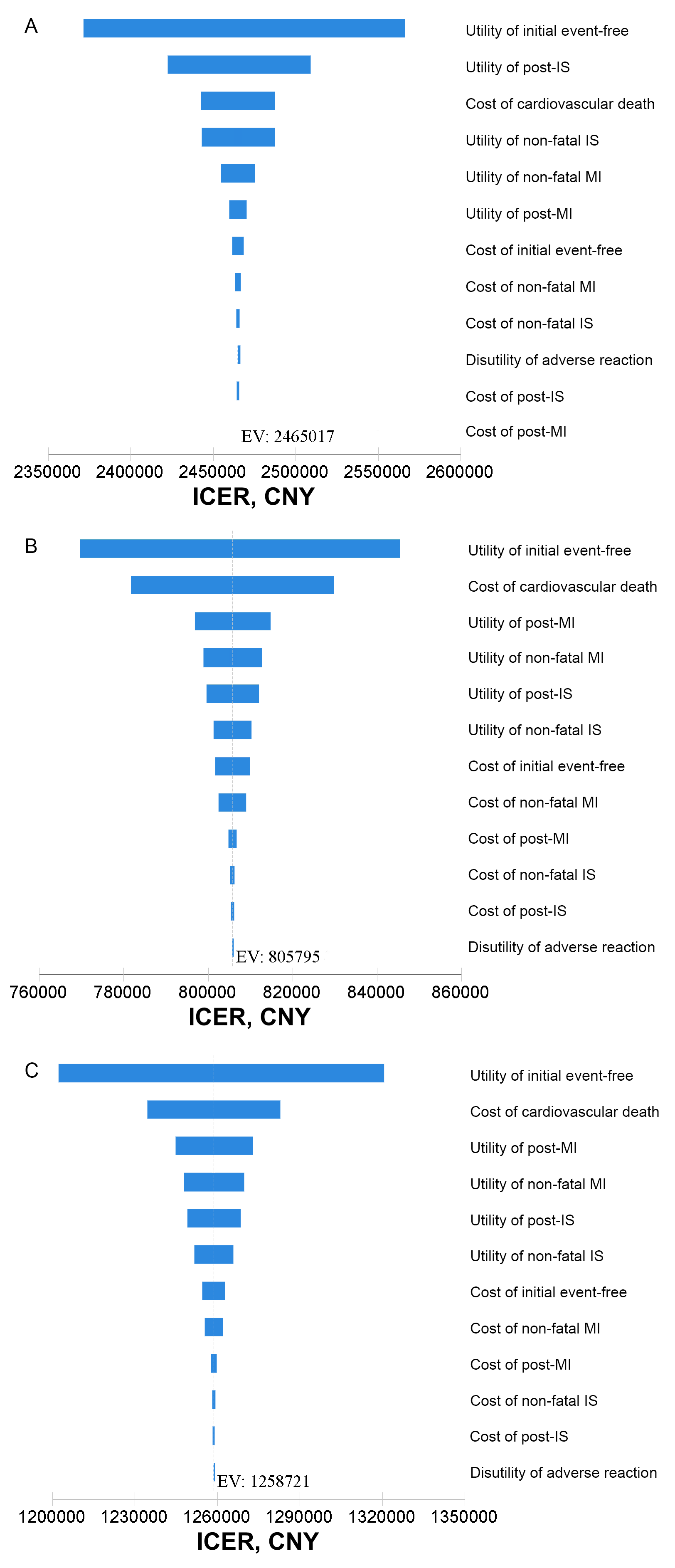
**

**(A)** Economic evaluation based on clinical follow-up efficacy at full price of alirocumab. **(B)** Economic evaluation based on LDL-C reduction at discounted price of alirocumab. **(C)** Economic evaluation based on LDL-C reduction at full price of alirocumab. CNY: Chinese Yuan; EV: expected value; ICER: incremental cost-effectiveness ratio; IS: ischemic stroke; MI: myocardial infarction.

**Figure S2 Monte Carlo simulation scatters plot**

**
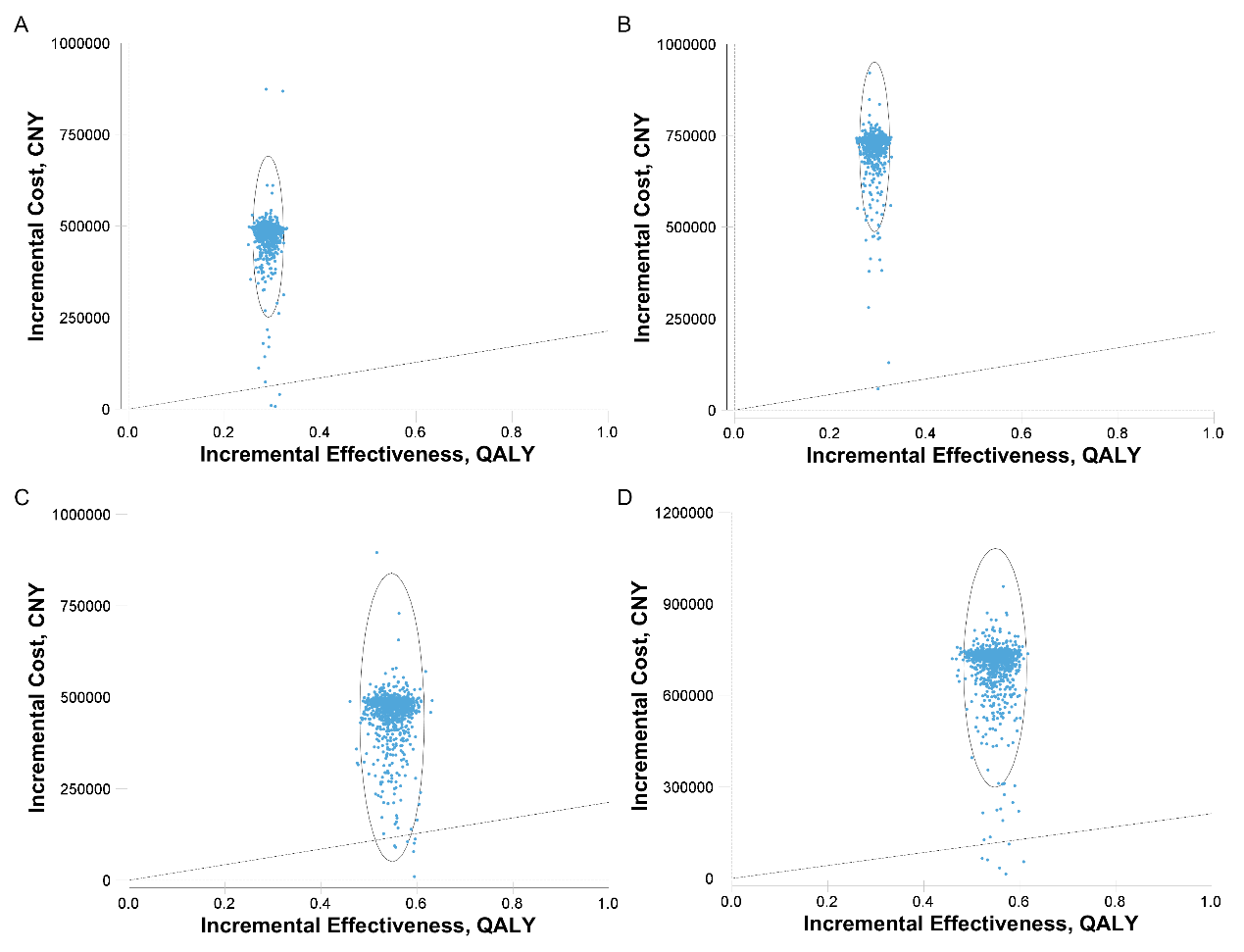
**

The ellipse shows the 95% confidence intervals, and the dotted line shows the willingness-to-pay threshold with a slope of 212,676 CNY per quality-adjusted life-year gained. **(A)** Economic evaluation based on clinical follow-up efficacy at discounted price of alirocumab. **(B)** Economic evaluation based on clinical follow-up efficacy at full price of alirocumab. **(C)** Economic evaluation based on LDL-C reduction at discounted price of alirocumab. **(D)** Economic evaluation based on LDL-C reduction at full price of alirocumab. CNY: Chinese Yuan; QALY: quality-adjusted life-year.
